# Supplementary material for: Multiscale red blood cell hitchhiking for targeted deep tissue gene delivery in lungs
Source: Nat Commun. 2025 Nov 21;16:10280. doi: 10.1038/s41467-025-65185-1 (PMC12638801; doi:10.1038/s41467-025-65185-1)
Supplement: Supplementary file 1 — Supplementary Information [file 41467_2025_65185_MOESM1_ESM.pdf]

Multiscale Red Blood Cell Hitchhiking for Targeted Deep Tissue Gene Delivery in Lungs

Kyung Soo Park,[1,2] Vineeth Chandran Suja,[1,2] Jayoung Kim,[1,2] Danika Rodrigues,[1,2] Malini Mukherji,[1,2] Maithili Joshi,[1,2] Yongsheng Gao,[1,2] Michael Griffith Bibbey,[1,2] Jeong-Won Choi,[1,2] Rick Liao,[1,2] Morgan E. Janes,[1,2,3] Andrés Da Silva Candal,[4] David L. Cameron,[5,6] Julian A.N.M. Halmai,[5,6] Kyle D. Fink,[5,6] Samir Mitragotri,[1,2,*] and Bijay Singh[1,2,*]

[1]John A. Paulson School of Engineering and Applied Sciences, Harvard University, Cambridge, MA, USA
[2]Wyss Institute of Biologically Inspired Engineering, Harvard University, Boston, MA, USA
[3]Harvard-MIT Division of Health Sciences and Technology, Massachusetts Institute of Technology, Cambridge, MA, USA
[4]Clinical Neurosciences Research Laboratory, Clinical University Hospital, Health Research Institute of Santiago de Compostela, Santiago de Compostela, Spain
[5]Neurology Department, Stem Cell Program and Gene Therapy Center, UC Davis Health System, Sacramento, CA, USA
[6]MIND Institute, UC Davis Health System, Sacramento, CA, USA

*Corresponding authors
Samir Mitragotri (mitragotri@seas.harvard.edu)
Bijay Singh (bijaysingh@seas.harvard.edu)

Supplementary Information

Table of Contents

SUPPLEMENTARY METHODS 2
SUPPLEMENTARY INFORMATION 3
SUPPLEMENTARY FIGURES 5
SUPPLEMENTARY TABLE 30
REFERENCES 31

Supplementary methods

Rheology study

For the rheological characterization, we measured the viscosity of RBC solutions with or without adding tannic acid (TA) and Fe using a TA Instruments HR 20 Discovery Hybrid rheometer fitted with a Peltier stage set to 25°C. Measurements were performed using a 20mm 2° conical plate geometry and a solvent trap to prevent sample drying during testing. Flow sweeps were performed on samples (n = 5) at shear rates between 1 to 1000 s^{-1} with five points per decade, and plotted as mean +/- SEM.

Preparation of LNP

LNP was prepared based on our previous report (*1*). Briefly, powdered lipids (DSPC, DMG-PEG, and cholesterol) were weighed and dissolved in pure ethanol to make 5 mg/mL stocks of each lipid. These stocks were then mixed with (6Z,9Z,28Z,31Z)-heptatriacont-6,9,28,31-tetraene-19-yl4-(dimethylamino)butanoate (DLin-MC3-DMA) to give a final DLin-MC3-DMA:cholesterol:DSPC: DSPE-Cy7: DMG-PEG molar ratio of 50:34.65:3.85:10:1.5. Lastly, the final total lipid concentration was adjusted to 6.25 mM by adding ethanol.

Luciferase mRNA (L-7202; TriLink BioTechnologies) was prepared at 55.8 µg/mL in 100 mM citrate buffer pH 4, mixed with the lipid stocks, and loaded into syringes. Precision Nanosystems NanoAssemblr Benchtop and/or Ignite was used to flow the mixtures through a microfluidic device for LNP synthesis. The resulting LNP had an average hydrodynamic diameter of 88 nm, PDI<0.02, and a zeta potential average of -30 mV.

Cryosectioning & imaging of tissues

After CO_2 euthanasia, mice were perfused with 10-20 mL of PBS, and then the lungs were extracted and stored in PBS containing 1% FBS. The lungs were submerged in OCT compound (Sakura) inside mini cassettes and stored at -80 °C overnight. Samples were sectioned with a cryostat (Thermo Scientific) to a 10 µm thickness, mounted onto a slide-glass (Fisherbrand, FIS 1255015), and stored at -80 °C until further processing. For staining, samples were brought to room temperature for 30 min, fixed with acetone at -20 °C for 10 min, and then placed back at room temperature for 10 min to dry the acetone. Next, the samples were washed with PBS, blocked with a blocking buffer (10% goat serum and 1% BSA in PBS) for 1 hour at room temperature, and stained with an antibody cocktail prepared by mixing the antibodies listed in **Table S1**.

The resulting samples were washed with PBS twice, then covered with a cover slip using DAPI-containing mounting media (Invitrogen, P36961). They were stored at room temperature overnight until imaged with a fluorescence microscope (Axioscan, Zeiss).

Flow cytometry gating strategy

Gating on endothelial (CD31) and epithelial (CD326) cells for flow cytometry was performed using previously reported gating strategies for these two cell types (*2, 3*). A detailed gating scheme is shown in **Supplementary Fig. 25**.

Supplementary information

Supplementary Information 1. Overview of the master targeting equation. The drug flux, J, across a vascular membrane with a permeability, P_{vas}, due to a concentration difference of Δc, can be expressed from Fick's law as,

$$J = P_{vas}\Delta C.$$

Under the following assumptions,
(a) The concentration of the drug on the exterior of the vasculature (in the tissue of interest) remains approximately 0 (or is much smaller than the concentration in blood),
(b) Drug distribution and clearance follows a single-compartment PK model, and
(c) Drug concentration in blood changes solely due to clearance (i.e., clearance rates are much higher than tissue adsorption rates),
The flux can connect to vascular drug concentration evolution analytically, giving the *master targeting equation* as follows:

$$J = C_o \lambda_{tis} P_{vas} e^{-\frac{t}{\tau}} \tag{1}$$

J, drug flux into tissues from blood circulation is a function of the initial drug concentration in blood upon injection (C_o), the enhancement of local tissue concentration of the drug achieved by the targeting strategy (λ_{tis}), the vascular endothelial permeability in the target tissue (P_{vas}), and the time constant of blood clearance of the drug (τ).

In the case of RBC hitchhiking, λ_{tis} and τ can be depicted as λ_{RBC} and τ_{RBC}, where λ_{RBC} is the amplification in local concentration at the target tissue due to the drug being loaded on the surface of RBC relative to free drug, and τ_{RBC} is the half-life of drugs loaded on RBC, giving the equation below (2):

$$J = C_0 P_{vas} \lambda_{RBC} e^{-\frac{t}{\tau_{RBC}}}. \tag{2}$$

This equation can be plotted into graphs with several example values for P_{vas}, λ_{RBC}, and τ_{RBC} as shown in **Supplementary Fig. 1**. Drug flux into tissue (**Supplementary Fig. 1A and 1C**) and cumulative area under the curve (**Supplementary Fig. 1B and 1D**) are plotted against time at a fixed C_o using P_{vas} of 1 and 2, λ_{RBC} of 1 and 3, and τ_{RBC} of 1 and 2. Higher drug flux and consequent higher drug accumulation are achieved with higher P_{vas}, λ_{RBC}, *and* τ_{RBC}.

Supplementary Information 2. Theoretical improvement in drug flux into tissue based on specific values. The total accumulated drug mass in the tissue can be obtained by recognizing $J = \frac{1}{A_{vas}}\frac{dQ}{dt}$, and integrating the above equation with respect to time, yielding,

$$Q = c_0 A_{vas} \tau_{RBC}\, P_{vas} \lambda_{RBC}$$

Here, Q is the amount of drug accumulated, A_{vas} is the area of vasculature. The enhancement in drug accumulation for RBC hitching with VEGF as compared to the current state of the art can be obtained as,

$$\frac{Q_{RBC,VEGF}}{Q} = \frac{\tau_{RBC}}{\tau}\frac{R_{vas}}{R}\lambda_{RBC}. \qquad (3)$$

The following geometrical and RBC-migration argument can be made to estimating λ_{RBC}.

RBC squeezing through a capillary of radius R with a gap ΔR, traps a volume per unit length of $2\pi R \Delta R$ (**Supplementary Fig. 24A**). The mass of drugs released from RBCs is distributed in this smaller volume rather than a volume per unit length $2\pi R^2$ of a large blood vessel. Assuming perfect drug detachment from the surface, this implies, we can expect an enhancement in drug concentration of the order of $\lambda_{RBC} \sim \frac{R}{\Delta R}$, which is about 10 based on known RBC hemodynamics (*4*).

In larger blood vessels, RBCs migrate away from the vascular surface due to shear-induced lift forces (*5*). As a result, the gap size increases, and shear-induced detachment decreases. Consequently, λ_{RBC} is expected to have a low value (at or below 1).

Compared to the traditional case, where we assume *P, λ,* and *τ* as (1, 1, 1), 2 fold increase in vascular permeability achieved by VEGF (P_{vas}), 2 fold increase in the availability of AAV in circulation (τ_{RBC}), and 10-fold increase in local tissue accumulation of AAV lead to significantly higher and durable drug flux into tissue (**Supplementary Fig. 24B**), and overall 40-fold increased accumulation of AAV at the target tissue (**Supplementary Fig. 24C**), based on the master targeting equation (1).

Putting $\frac{\tau_{RBC}}{\tau} \approx 2$ and $\frac{P_{vas}}{P} \approx 2$ into equation (3), $\frac{Q_{RBC,VEGF}}{Q}$ is expected to be approximately equal to 40, matching the enhancement value from above.

Supplementary figures

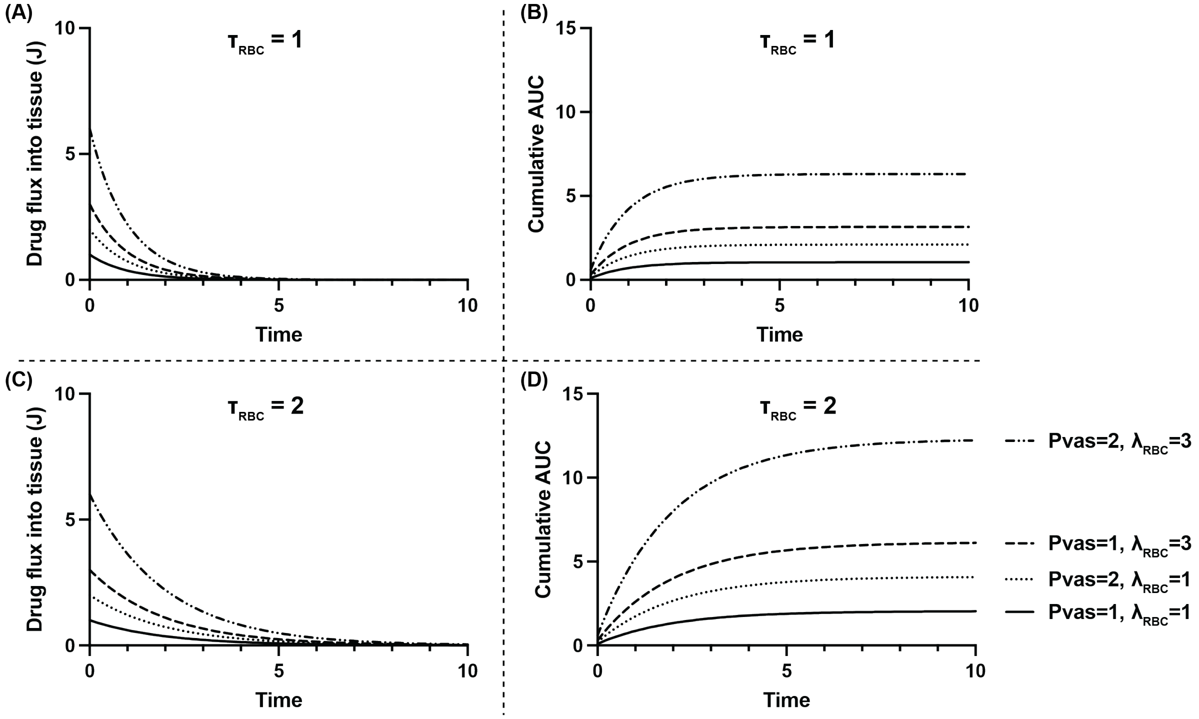

Supplementary Figure 1. Analytical modeling of drug flux into tissue with red blood cell (RBC) hitchhiking. Drug flux into tissue (**A and C**) and cumulative area under the curve (AUC, **B and D**) are shown as a function of time based on the master targeting equation.

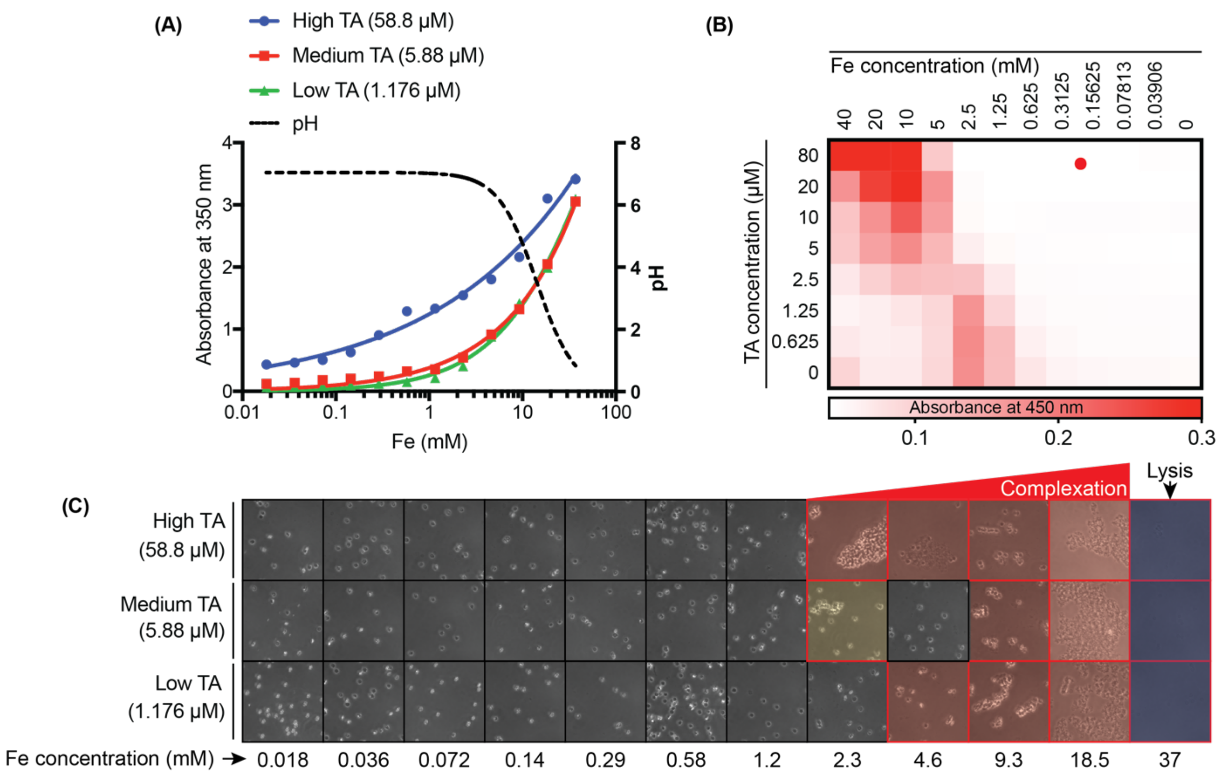

Supplementary Figure 2. Formulation optimization to minimize hemolysis and aggregation. (**A**) Aggregation between Fe and TA was measured by absorbance at 350 nm. Aggregation was prevented at Fe concentrations below 1 mM, where the pH was maintained neutral. (**B**) Hemolysis was measured by absorbance at 450 nm of the supernatant of mixtures of RBC, TA, and Fe at different concentrations. The red dot indicates the condition with which the final formulation is made. (**C**) Aggregation was assessed under a light microscope by mixing RBC, TA, and Fe at different concentrations.

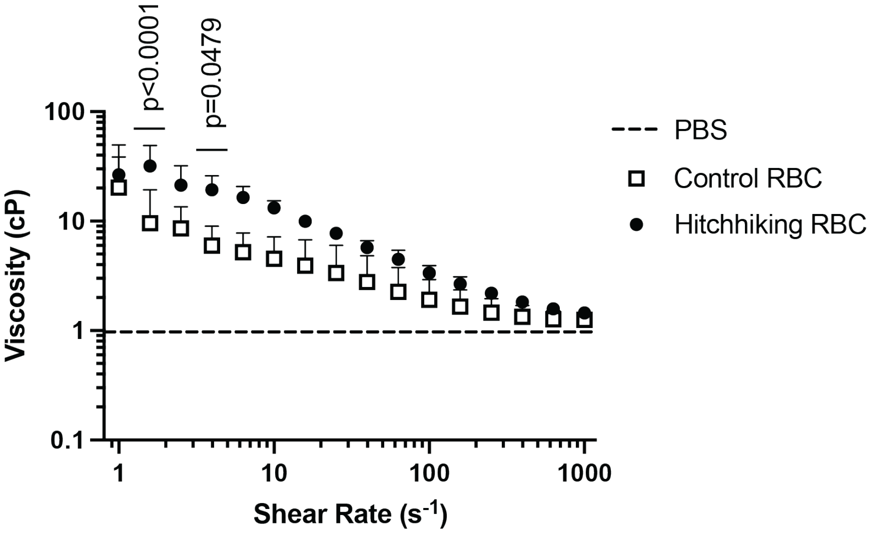

Supplementary Figure 3. Viscosity of RBC at 10% hematocrit. Measured with a rheometer (HR20; TA Instruments). Independent biological replicates of n=3 for PBS, n=6 for Control RBC, and n=5 for Hitchhiking RBC. Error bars represent mean ± SEM. Statistical analysis was done using a two-way ANOVA, followed by Sidak's multiple comparisons test.

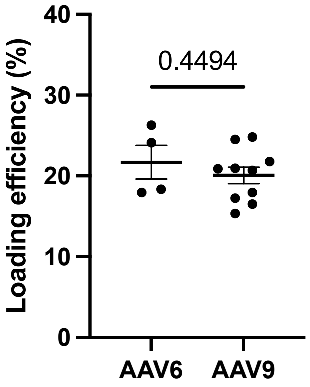

Supplementary Figure 4. Loading efficiencies of AAV6-CMV-Luc and AAV9-CMV-dsRED on RBCs. The loading condition described in the methods section was used. Data are consolidated from three independent experiments (independent biological replicates of n=4 for AAV6 and n=10 for AAV9). Error bars represent mean ± SEM. An unpaired two-sided Student's t-test was used for statistical analysis.

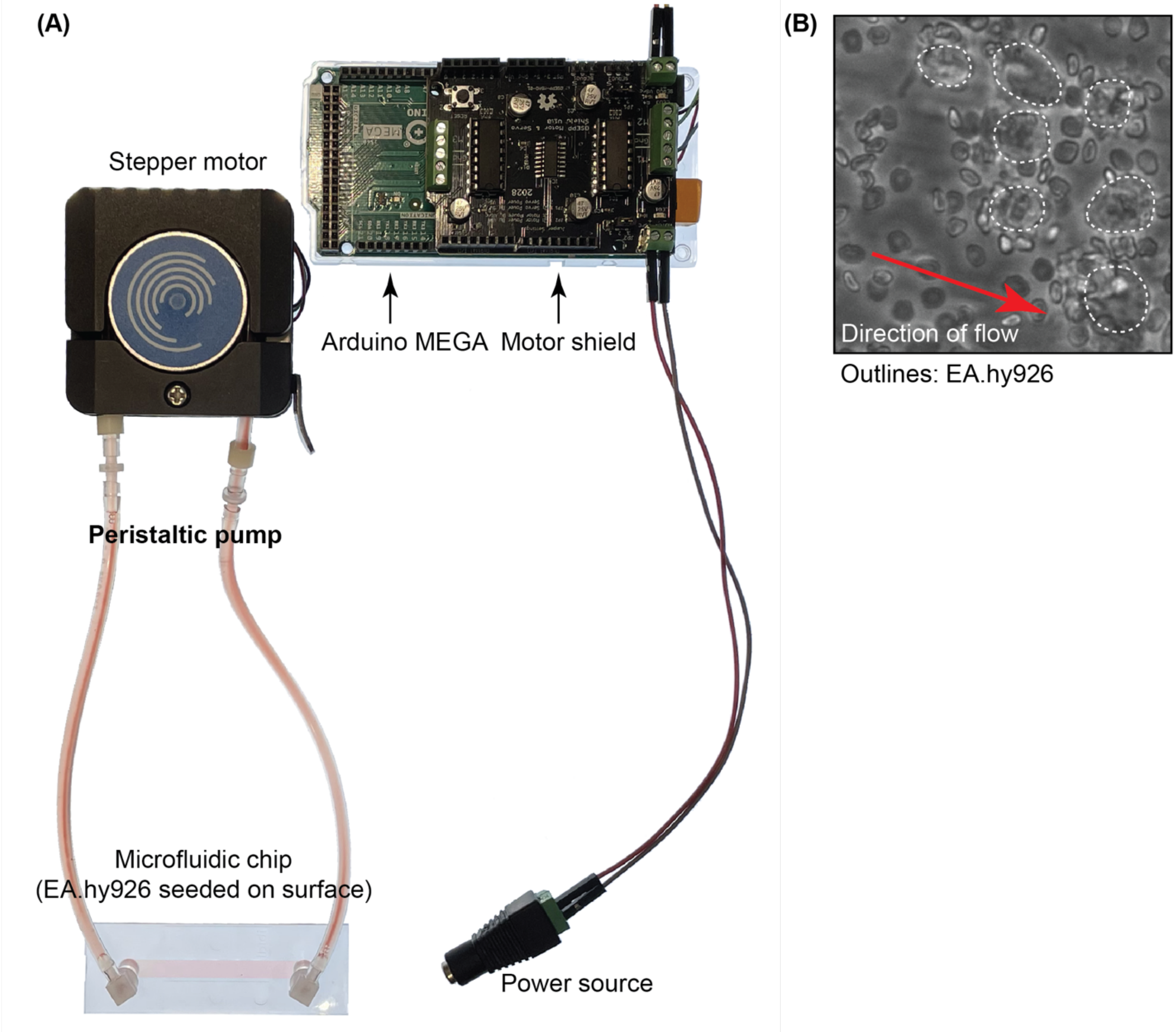

Supplementary Figure 5. Setup for the in vitro binding study with flow. (**A**) A peristaltic pump, consisting of a stepper motor and a power system, was used to continuously flow either PBS or cell culture media into a microfluidic chip. EA.hy926 human endothelial cell line was seeded at the surface of the chip as a model endothelium, on top of which AAV-loaded RBC is flown. (**B**) The microfluidic chip was monitored with a microscope to visually confirm the flow of RBC on the model endothelial cells. Representative image from two independent experiments.

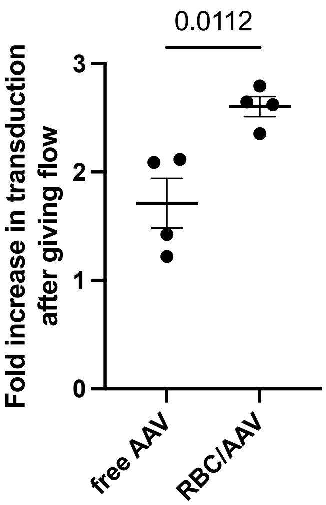

Supplementary Figure 6. Flow enhances transduction in the model endothelial cell line EA.hy926 by AAVs. The fold changes in transduction between static and flow conditions were measured 2 weeks after treating EA.hy926 cells with AAV encoding luciferase (AAV6-CMV-luc) in microfluidic chips. Luminescence was measured using a plate reader. n=4 independent biological replicates. Error bars represent mean ± SEM. Statistical analysis was performed with an unpaired two-sided Student's t-test.

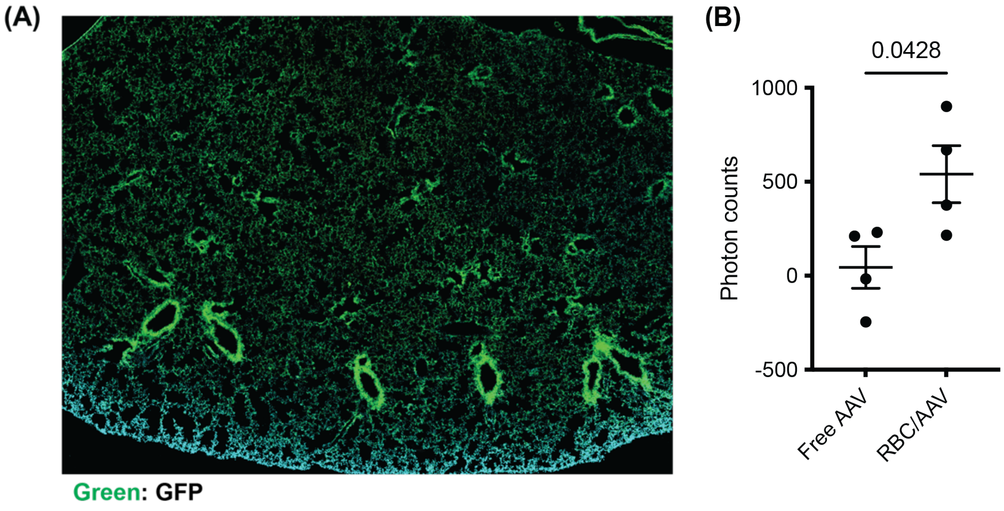

Supplementary Figure 7. Transgene expression in the lungs. (**A**) Lungs of mice treated with RBC/AAV using GFP-encoded AAV (AAV6-CMV-GFP) were harvested three weeks after intravenous injection via the tail vein for cryosectioning and fluorescence imaging. Representative image from two independent experiments. (**B**) GFP transgene expression in the lungs of mice treated with free AAV or RBC/AAV was measured 12 weeks after injection via ex vivo IVIS imaging (n = 4 female C57BL/6 mice per group, 5-6 weeks of age). Error bars represent mean ± SEM. Statistical analysis was performed with an unpaired two-sided Student's t-test.

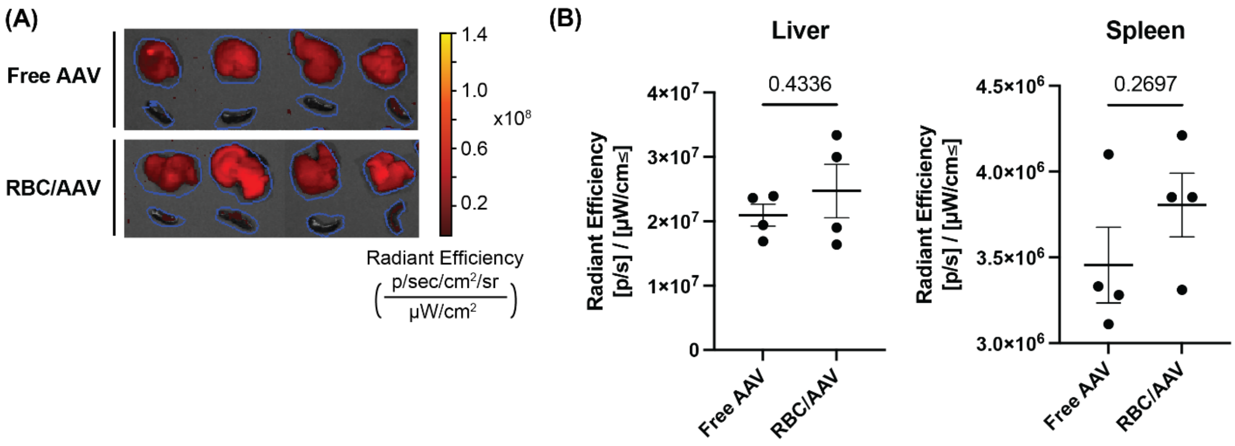

Supplementary Figure 8. Transgene expression in the liver and spleen 21 days after CA injection of 1.51e+10 vg AAV9-CMV-dsRED formulations. (**A**) *Ex vivo* images of the livers and spleens. Representative images from two independent experiments. (**B**) Average radiant efficiency of dsRED signals from the liver and spleen. n=4 female C57BL/6 mice per group, 10 weeks of age. Error bars represent mean ± SEM. An unpaired two-sided Student's t-test was performed for statistical analyses.

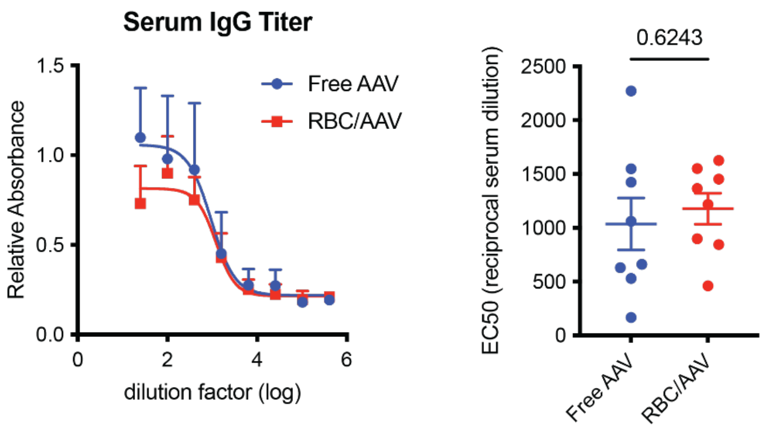

Supplementary Figure 9. Serum IgG titer of free AAV or RBC/AAV-injected mice. Two IV injections were given on days 0 and 28, followed by serum collection on day 56. ELISA against mouse total IgG was performed on serially diluted sera (**left**). EC50 (reciprocal serum dilution) was used to quantify the serum IgG titers (**right**). n=8 female C57BL/6 mice per group, 5-6 weeks of age. Error bars represent mean ± SEM. An unpaired two-sided Student's t-test was used for statistical analysis.

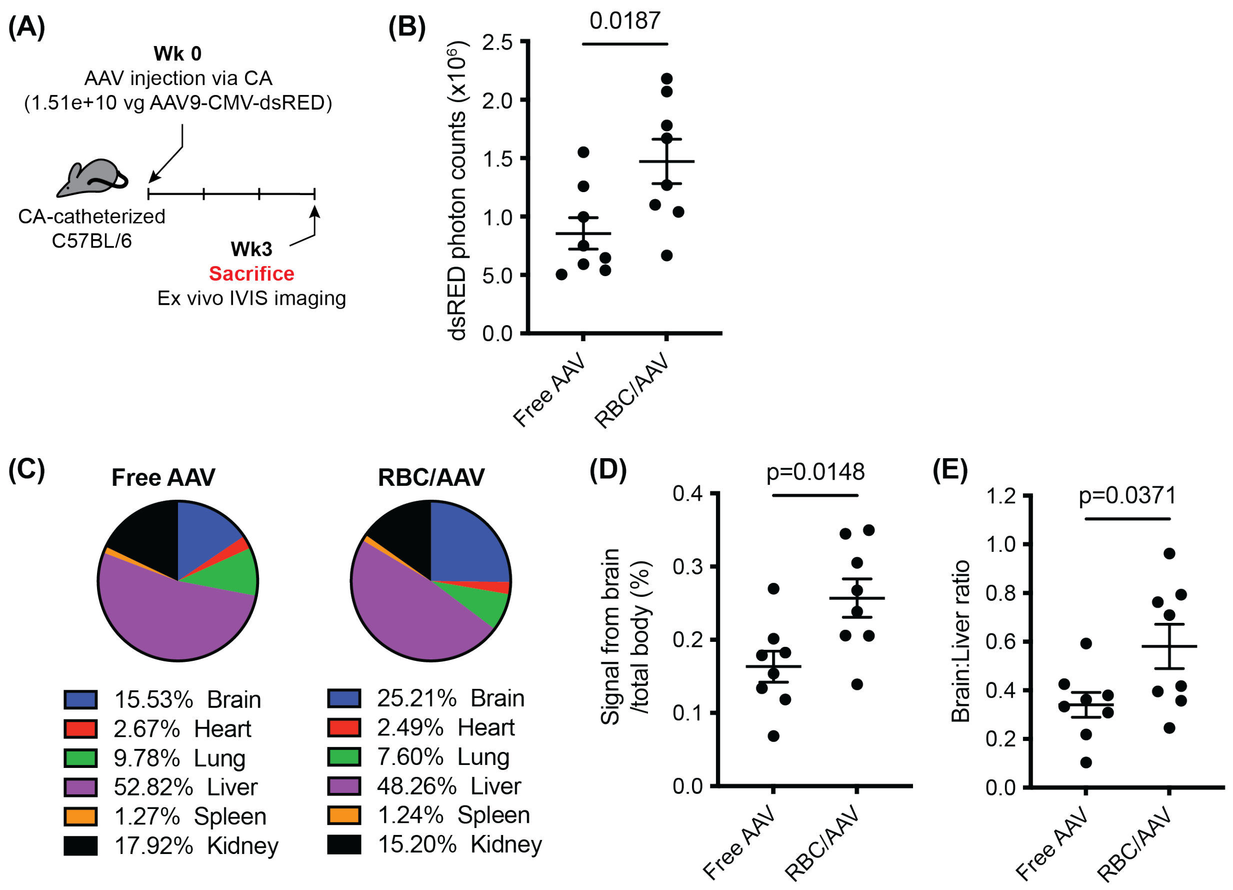

Supplementary Figure 10. Transgene expression in the brain via carotid arterial injection of AAV. (**A**) Timeline of the experiment. 1.51e+10 vg of AAV9 encoding dsRED (AAV9-CMV-dsRED) was intra-arterially injected via the carotid artery. n=8 female C57BL/6 mice per group, 5-6 weeks of age, were sacrificed three weeks after injection for *ex vivo* IVIS imaging. (**B**) Transgene expression in the brain was quantified by measuring the dsRED signals from the brain using IVIS. (**C**) Percent distribution of expressed transgene signals (dsRED) from organs. (**D**) Corresponding portion of brain signals from the total body and (**E**) brain-to-liver ratios. The presented data are consolidated data from two independent experiments. Error bars represent mean ± SEM. Statistical analysis was performed with an unpaired two-sided Student's t-test.

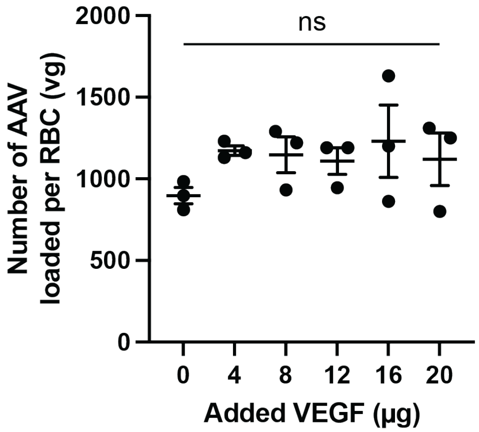

Supplementary Figure 11. Number of AAVs loaded per RBC by the added amount of VEGF during MARVEL formulation, measured by PCR. n=3 independent biological replicates. Error bars represent mean ± SEM. Statistical analysis was performed with a one-way ANOVA. ns: not significant.

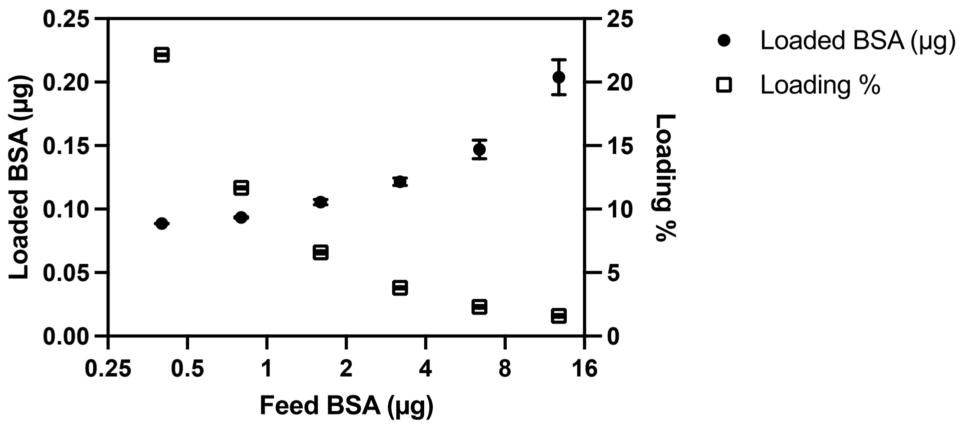

Supplementary Figure 12. FITC-BSA loading on RBC. FITC-BSA loading was measured using a plate reader with a standard curve prepared with a mixture of FITC-BSA and TA. The loading trend showed a dose-dependent increase. n=6 independent biological replicates. Error bars represent mean ± SEM.

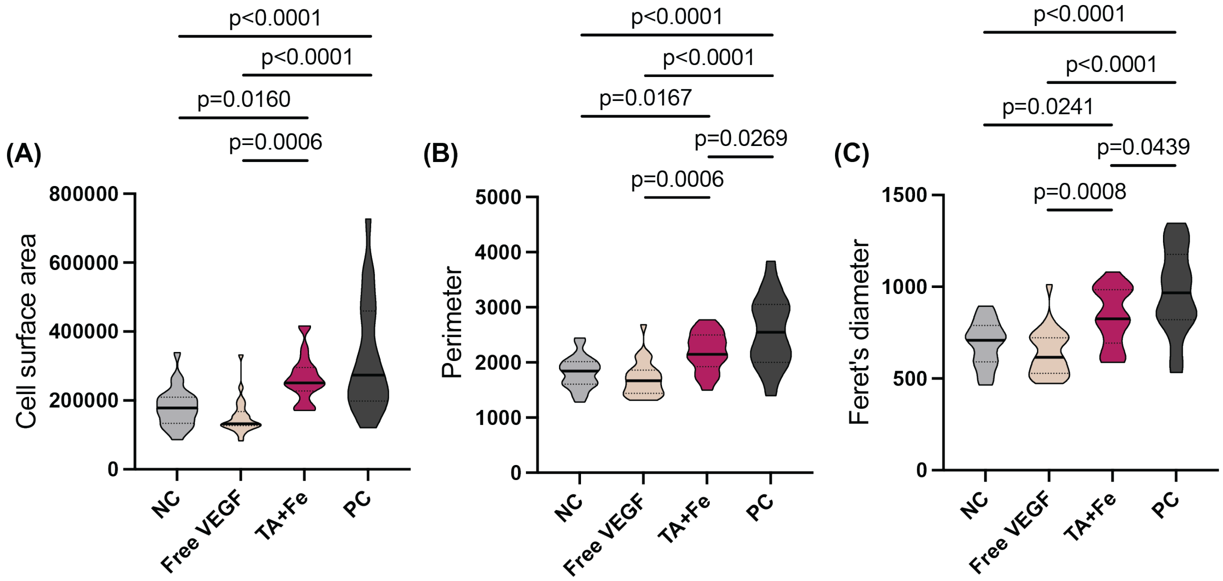

Supplementary Figure 13. Morphological analysis of primary human brain endothelial cells after VEGF treatment. NC, negative control. PC, positive control. No treatment was done on NC. For PC, 100 ng/mL of VEGF was treated for 24 hours. For the free VEGF and TA+Fe groups, 20 ng/mL of VEGF was treated for 6 hours. The resulting cells were stained for confocal imaging. (**A**) The cell surface area, (**B**) perimeter, and (**C**) Feret's diameter were measured with Fiji software. Data are from n=7 (NC), n=5 (Free VEGF), and n=6 (VEGF+TA+Fe and PC) of independent planes on confocal images. Statistical analysis was performed with a one-way ANOVA, followed by Tukey's multiple comparisons test.

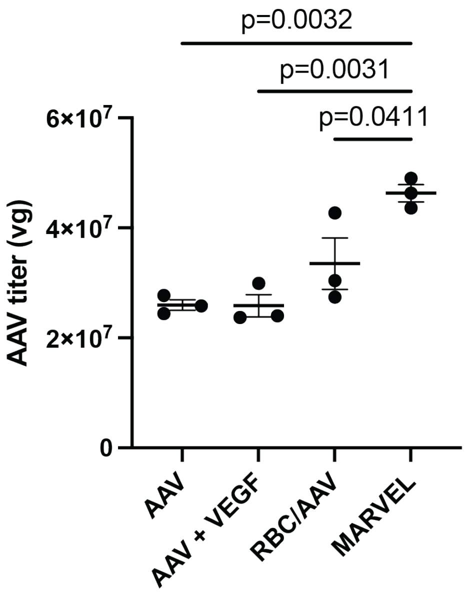

Supplementary Figure 14. Transwell setup using EA.hy926-coated insert for AAV transmigration across the endothelial layer. The transmigrated AAV in the bottom chamber after 48 hours of incubation was quantified with PCR. n=3 independent biological replicates. Error bars represent mean ± SEM. Statistical analysis was performed with a one-way ANOVA, followed by Tukey's multiple comparisons test.

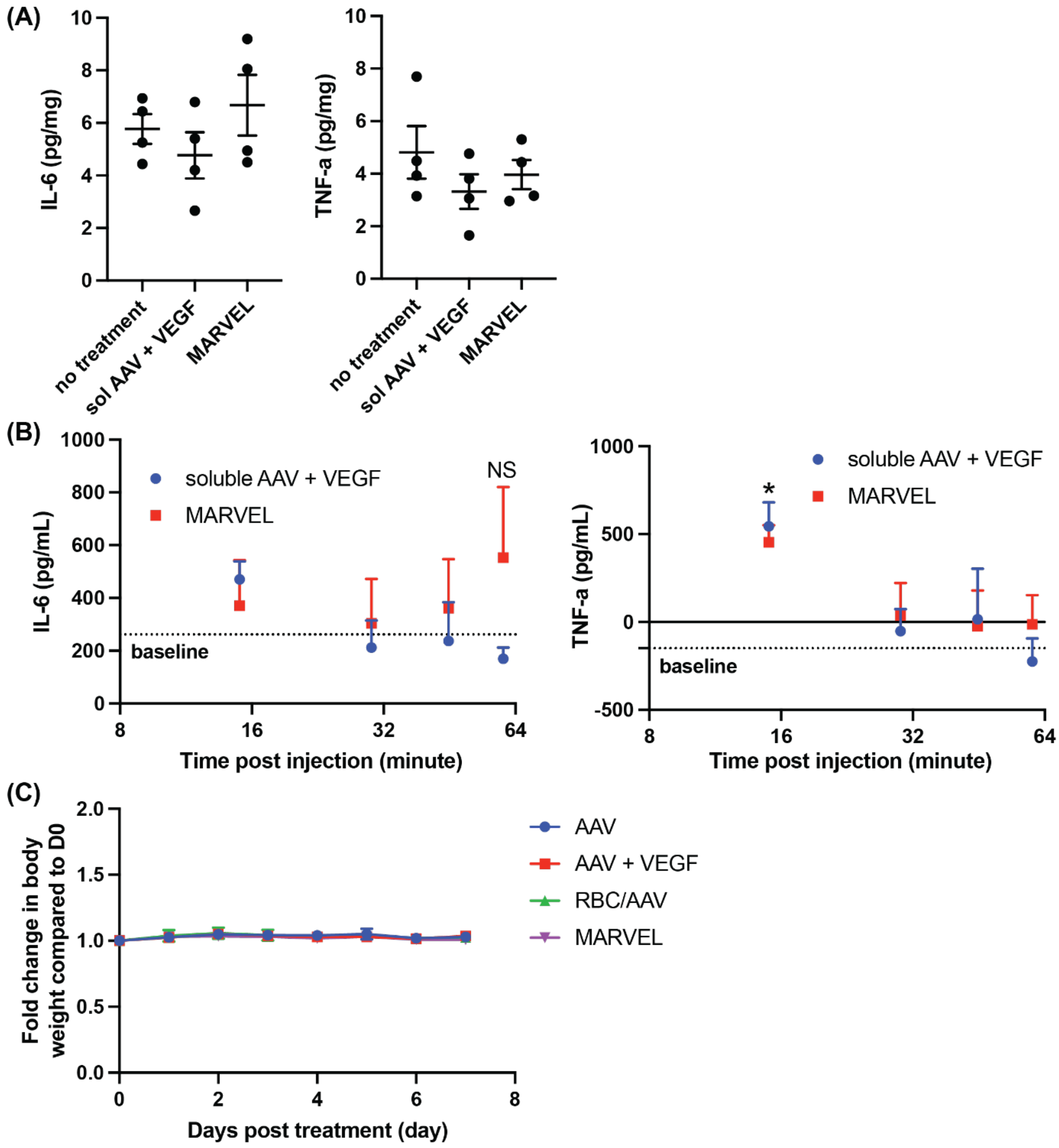

Supplementary Figure 15. In vivo safety study. IL-6 and TNF-α concentrations (**A**) in the lungs 4 hours after injection and (**B**) in the serum 15, 30, 45, and 60 minutes after injection were measured with ELISA (n=4 female C57BL/6 mice per group, 5-6 weeks of age). (**C**) Fold body weight change compared to the weights measured on D0 before treatment (n=5 female C57BL/6 mice per group, 5-6 weeks of age). Overall, the data indicate the safety of the formulation. Statistical analyses for A were done with one-way ANOVA. For B, one-way ANOVA was performed by comparing values at each time point to the baseline value.

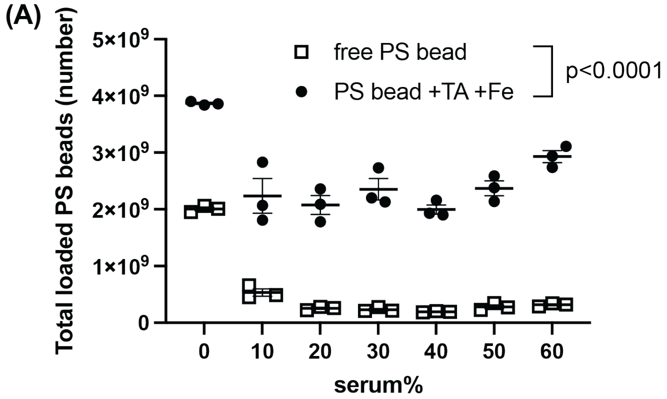

Supplementary Figure 16. Competition binding assay in varied serum concentrations. RBCs at 10% hematocrit in varying serum concentrations were mixed with 4.37e+10 fluorescent polystyrene (PS) beads with or without TA and Fe. Immediately after mixing, excess volume of PBS (5% BSA) was added to quench the reaction. n=3 independent biological replicates. (**A**) Total number of loaded PS beads by serum percentage. (**B**) Fold increases in loading with TA and Fe addition. n=3 independent biological replicates. Error bars represent mean ± SEM. (A) Two-way ANOVA, followed by Sidak's multiple comparisons test, was performed.

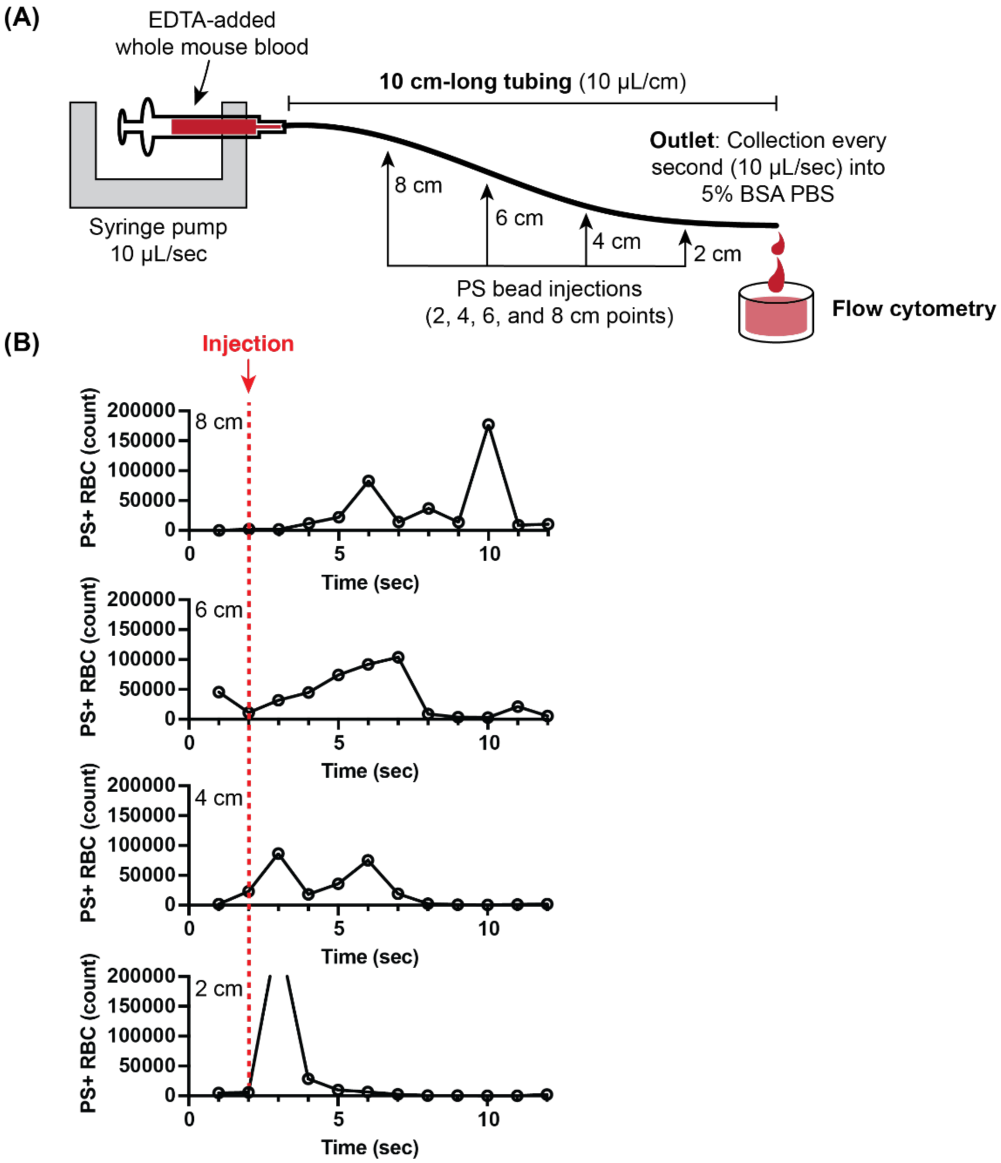

Supplementary Figure 17. Model drug loading on RBC in whole blood. (**A**) PS bead loading setup for evaluating the binding to RBC in flow. The mouse blood flow was given with a syringe pump at a 10 µL/sec rate into a 10 cm tubing with an inner diameter of 0.508 mm connected via a 25 gauge needle. PS beads were pulse-injected into different positions along the tubing (2, 4, 6, and 8 cm points), and samples were collected from the outlet every second. (**B**) Collected samples were run on a flow cytometer to assess the binding.

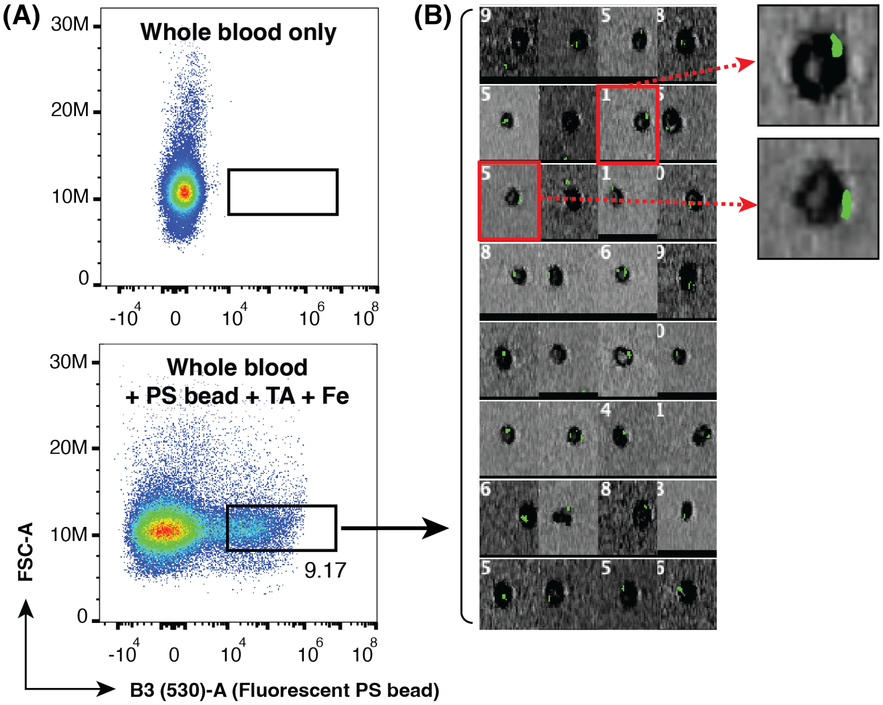

Supplementary Figure 18. Real-time imaging of model drug (PS beads) bound to RBC in whole blood. (**A**) Flow cytometry plots of neat whole mouse blood (upper) or pulse-mixed with PS beads, TA, and Fe (lower). (**B**) The bead-positive population was gated and imaged in real-time using a BD FACS Discover S8. n=3 independent biological replicates.

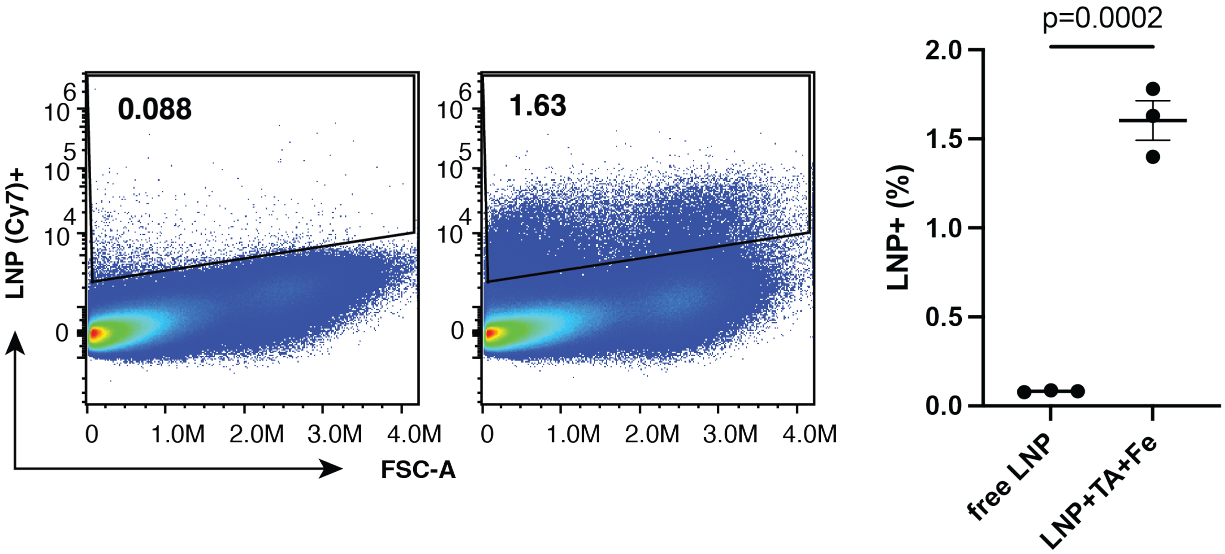

Supplementary Figure 19. Loading of Cy7-labeled lipid nanoparticle (LNP) on RBC. LNP containing a model mRNA (Luciferase mRNA) was mixed into whole mouse blood with or without TA and Fe in a static condition, and measured with a flow cytometer. n=3 independent biological replicates. Error bars represent mean ± SEM. Statistical analysis was performed with an unpaired two-sided Student's t-test.

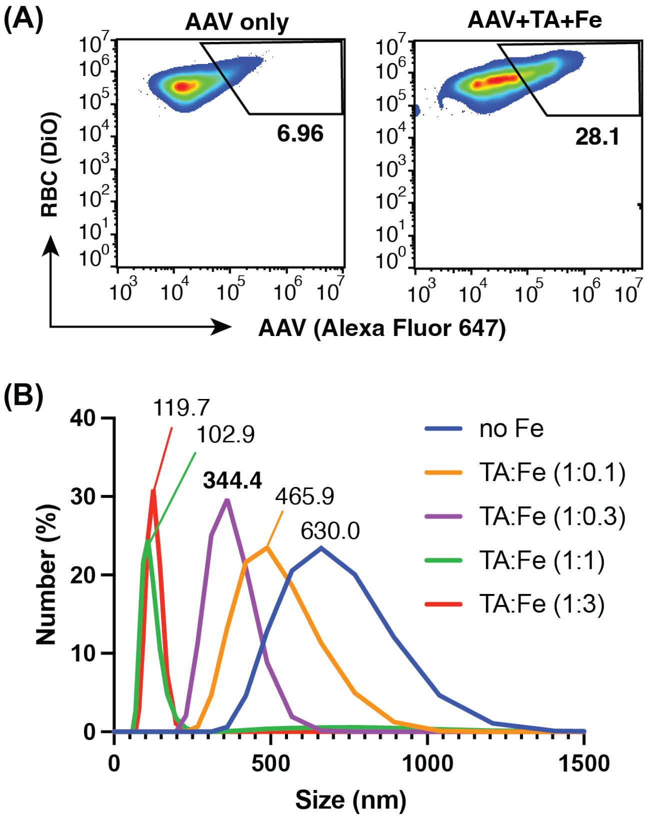

Supplementary Figure 20. Characterization of AAV, TA, and Fe complex. (**A**) Representative flow plots of AAV binding on RBC in whole blood with or without the addition of TA and Fe. (**B**) The sizes of complexes measured with dynamic light scattering (DLS) at different TA-to-Fe ratios. The current study used a TA-to-Fe ratio of 1:0.3, showing 344.4 nm of hydrodynamic size. AAV6-CMV-Luc was used. Representative plot obtained from n=3 independent biological replicates.

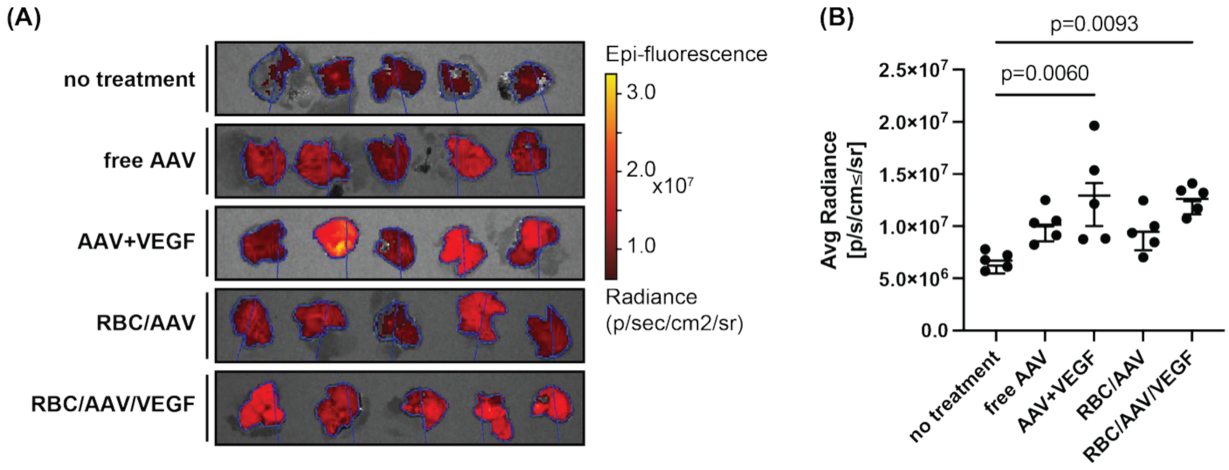

Supplementary Figure 21. Transgene expression in the liver 30 days after IV injection of AAV6-CMV-GFP formulations. (**A**) *Ex vivo* images of the livers. (**B**) Average radiance of GFP signals from the liver. n=5 female C57BL/6 mice per group, 5-6 weeks of age. Error bars represent mean ± SEM. One-way ANOVA, followed by Tukey's multiple comparisons test, was performed for (B).

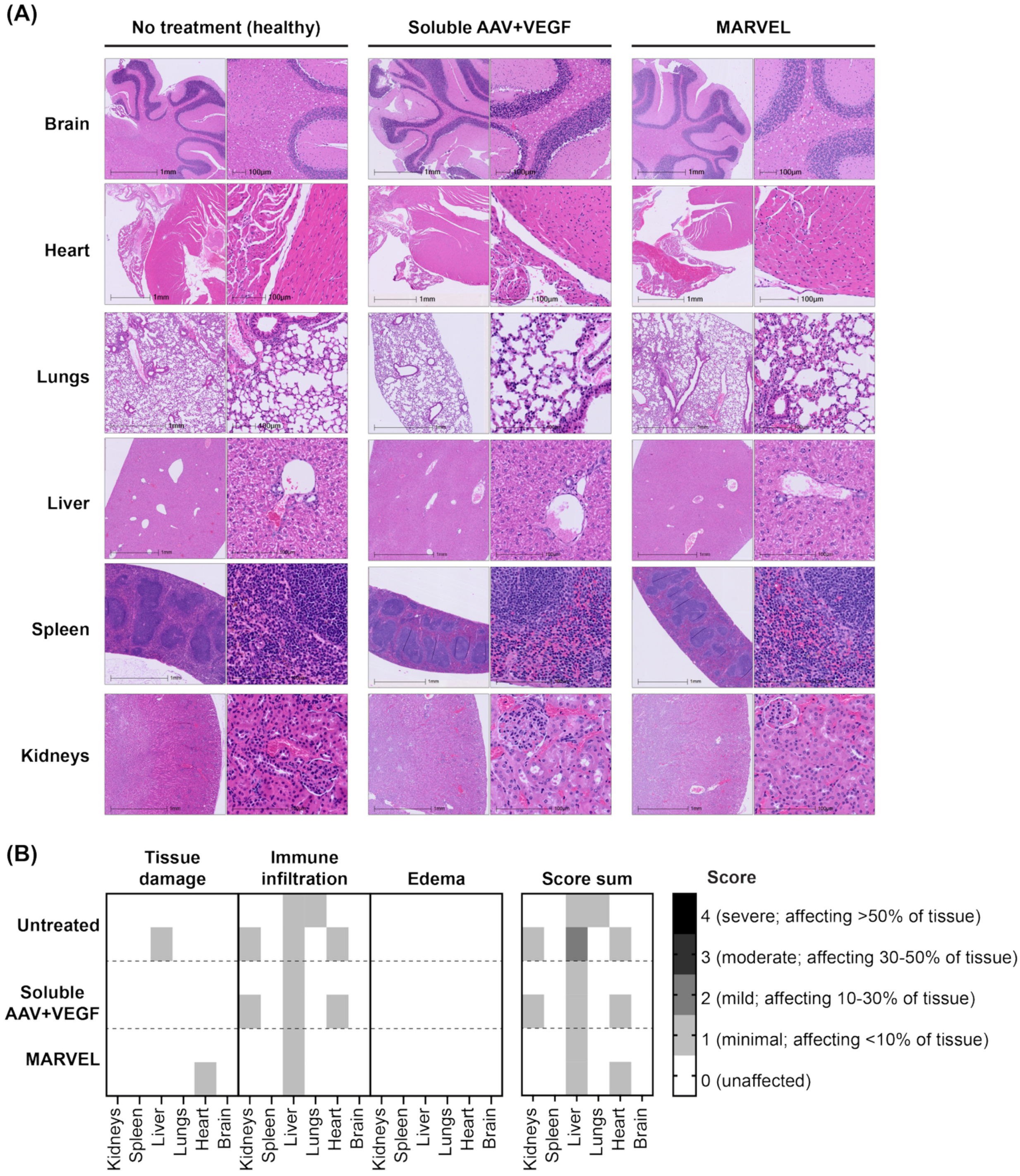

Supplementary Figure 22. Safety evaluation. (A) Histology sections of major organs harvested 30 days post-IV injection of *in situ* MARVEL formulation (AAV6-CMV-GFP). (**B**) Toxicities scored by a pathologist based on the histopathological images. Two mice from 5 mice (no treatment group) and 7 mice (soluble AAV+VEGF and MARVEL groups each) were randomly chosen for evaluation. Female C57BL/6 mice, 5-6 weeks of age, were used.

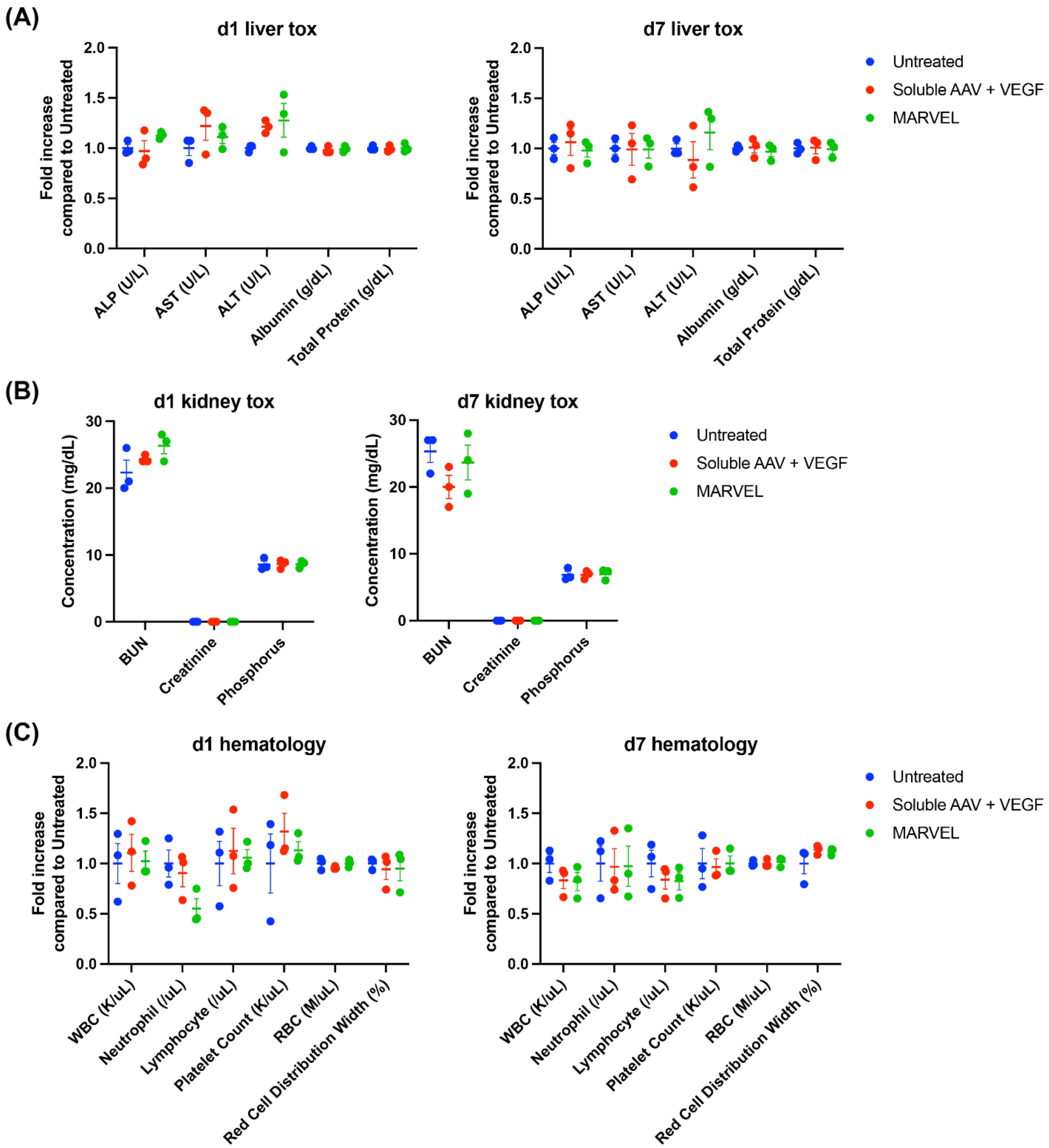

Supplementary Figure 23. Systemic toxicity assessment following intravenous administration of MARVEL formulation. (**A**) Liver toxicity markers, (**B**) Kidney function markers, and (**C**) Hematology parameters on days 1 (d1) and 7 (d7) post-treatment. n=3 female C57BL/6 mice per group, 5-6 weeks of age. Error bars represent mean ± SEM. One-way ANOVA was performed individually for each marker. No statistical significance was observed. ALP, alkaline phosphatase; AST, aspartate aminotransferase; ALT, alanine aminotransferase; BUN, blood urea nitrogen.

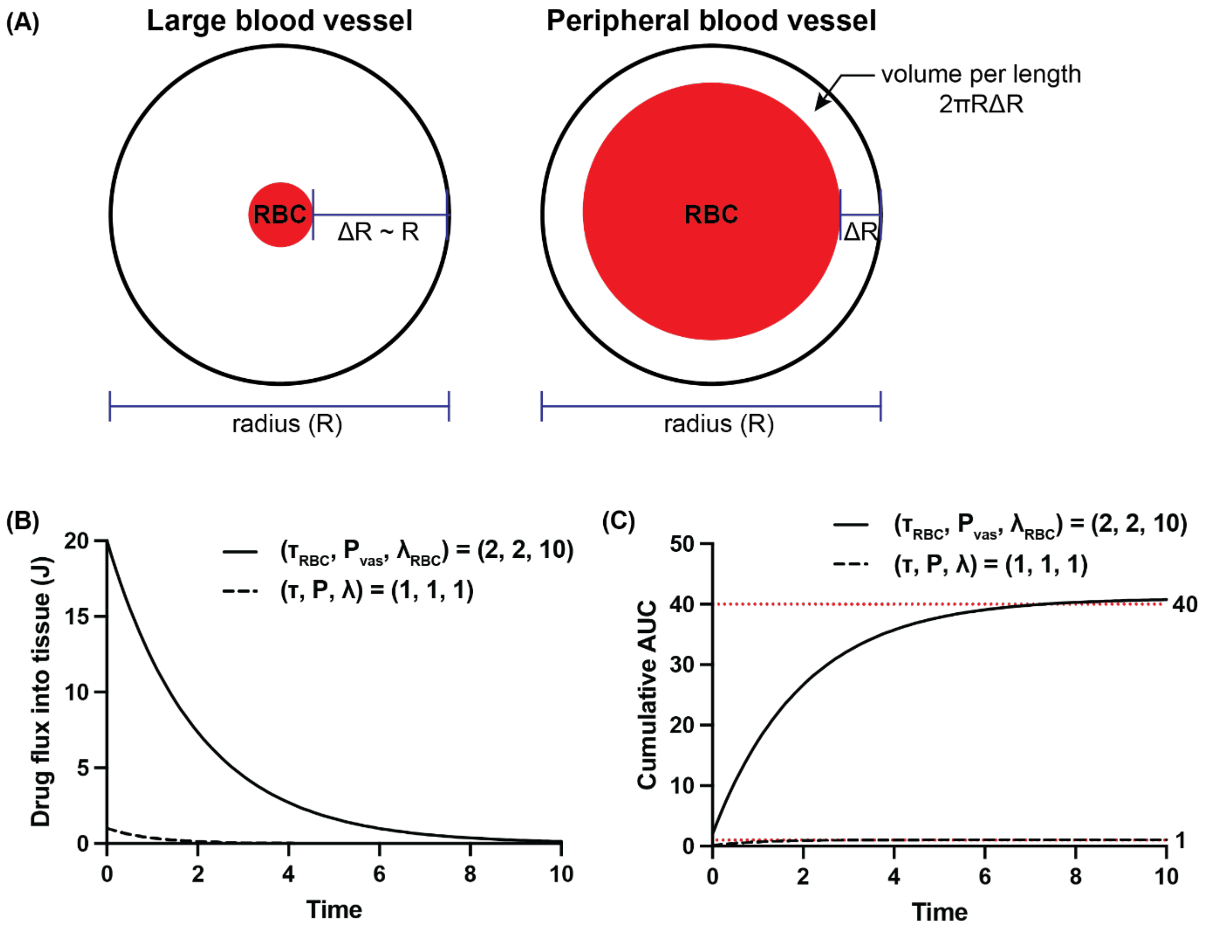

Supplementary Figure 24. Synergistic effects of vessel diameter, vascular permeability, and RBC hitchhiking on drug accumulation. (**A**) Schematic illustrating how vessel geometry influences local drug concentration. (**B**) Drug flux into tissue over time for enhanced vs. baseline parameters. (**C**) Cumulative drug accumulation (AUC), showing ~40-fold increase with RBC hitching and VEGF.

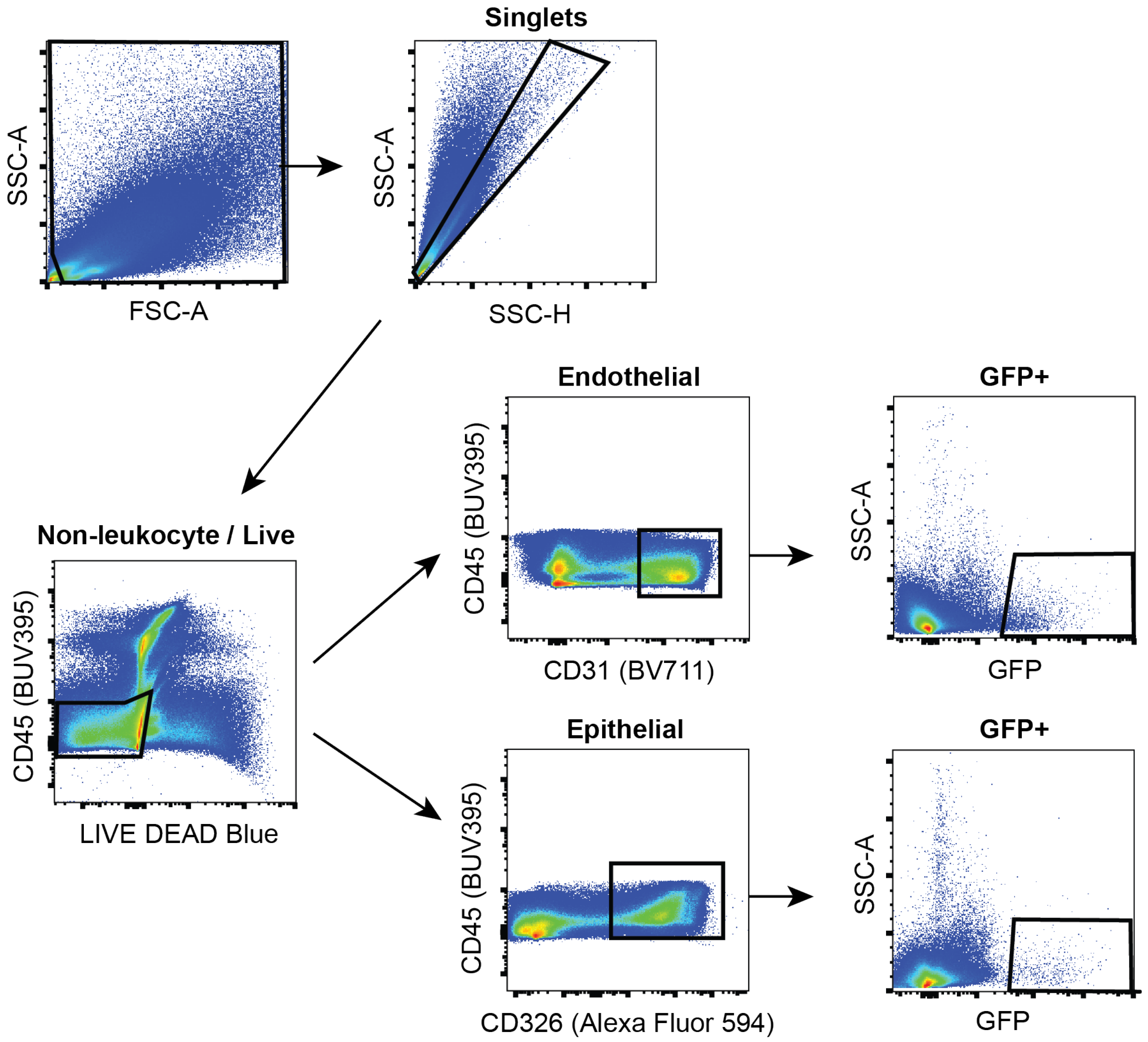

Supplementary Figure 25. Flow cytometry gating strategy for endothelial and epithelial cells. Cells were first gated based on forward and side scatter to exclude debris, followed by singlet selection. Live, non-leukocyte populations were identified using LIVE/DEAD Blue and CD45 (BUV395) staining. Endothelial cells were defined as CD31+CD45-, and epithelial cells as CD326+CD45-. GFP+ subsets were further identified within each population.

Supplementary table

Table S1. List of antibodies for tissue section staining.

Target	Fluor	Dilution	Manufacturer	Cat. No.
Rabbit anti-mouse GFP		1:200	Fabgennix	GFP-101AP
Goat anti-rabbit IgG	AF488	4 μg/mL	Invitrogen	A-11008

References

1. A. M. Curreri *et al.*, Localization of Intramuscular mRNA Delivery Using Deep Eutectic-Lipid Nanocomposites. *Adv Healthc Mater* **13**, e2400327 (2024).
2. S. Ghosh *et al.*, Enteric viruses replicate in salivary glands and infect through saliva. *Nature* **607**, 345-350 (2022).
3. F. Palumbo *et al.*, Impact of different tissue dissociation protocols on endothelial cell recovery from developing mouse lungs. *Cytometry A* **105**, 521-535 (2024).
4. T. W. Secomb, R. Hsu, A. R. Pries, Motion of red blood cells in a capillary with an endothelial surface layer: effect of flow velocity. *Am J Physiol Heart Circ Physiol* **281**, H629-636 (2001).
5. Z. Zhao *et al.*, Engineering of Living Cells with Polyphenol-Functionalized Biologically Active Nanocomplexes. *Adv Mater* **32**, e2003492 (2020).
