# Supplementary material for: Multiscale red blood cell hitchhiking for targeted deep tissue gene delivery in lungs
Source: Nat Commun. 2025 Nov 21;16:10280. doi: 10.1038/s41467-025-65185-1 (PMC12638801; doi:10.1038/s41467-025-65185-1)
Supplement: Supplementary file 2 — Reporting Summary [file 41467_2025_65185_MOESM2_ESM.pdf]

nature portfolio

Corresponding author(s): Samir Mitragotri
Bijay Singh

Last updated by author(s): Aug 28, 2025

Reporting Summary

Nature Portfolio wishes to improve the reproducibility of the work that we publish. This form provides structure for consistency and transparency in reporting. For further information on Nature Portfolio policies, see our Editorial Policies and the Editorial Policy Checklist.

Statistics

For all statistical analyses, confirm that the following items are present in the figure legend, table legend, main text, or Methods section.

n/a	Confirmed	
☐	☒	The exact sample size (*n*) for each experimental group/condition, given as a discrete number and unit of measurement
☐	☒	A statement on whether measurements were taken from distinct samples or whether the same sample was measured repeatedly
☐	☒	The statistical test(s) used AND whether they are one- or two-sided<br>*Only common tests should be described solely by name; describe more complex techniques in the Methods section.*
☐	☒	A description of all covariates tested
☐	☒	A description of any assumptions or corrections, such as tests of normality and adjustment for multiple comparisons
☐	☒	A full description of the statistical parameters including central tendency (e.g. means) or other basic estimates (e.g. regression coefficient) AND variation (e.g. standard deviation) or associated estimates of uncertainty (e.g. confidence intervals)
☐	☒	For null hypothesis testing, the test statistic (e.g. *F*, *t*, *r*) with confidence intervals, effect sizes, degrees of freedom and *P* value noted<br>*Give P values as exact values whenever suitable.*
☒	☐	For Bayesian analysis, information on the choice of priors and Markov chain Monte Carlo settings
☒	☐	For hierarchical and complex designs, identification of the appropriate level for tests and full reporting of outcomes
☒	☐	Estimates of effect sizes (e.g. Cohen's *d*, Pearson's *r*), indicating how they were calculated

Our web collection on statistics for biologists contains articles on many of the points above.

Software and code

Policy information about availability of computer code

Data collection	No software was used.
Data analysis	FlowJo 10.10.0 was used to analyze flow cytometry data. GraphPad Prism 10.5.0 was used for statistical analysis of data.

For manuscripts utilizing custom algorithms or software that are central to the research but not yet described in published literature, software must be made available to editors and reviewers. We strongly encourage code deposition in a community repository (e.g. GitHub). See the Nature Portfolio guidelines for submitting code & software for further information.

Data

Policy information about availability of data

All manuscripts must include a data availability statement. This statement should provide the following information, where applicable:

- Accession codes, unique identifiers, or web links for publicly available datasets
- A description of any restrictions on data availability
- For clinical datasets or third party data, please ensure that the statement adheres to our policy

The authors confirm that all data are available within the article, Supplementary Information, and Source Data files.

Research involving human participants, their data, or biological material

Policy information about studies with human participants or human data. See also policy information about sex, gender (identity/presentation), and sexual orientation and race, ethnicity and racism.

Reporting on sex and gender	*Use the terms sex (biological attribute) and gender (shaped by social and cultural circumstances) carefully in order to avoid confusing both terms. Indicate if findings apply to only one sex or gender; describe whether sex and gender were considered in study design; whether sex and/or gender was determined based on self-reporting or assigned and methods used. Provide in the source data disaggregated sex and gender data, where this information has been collected, and if consent has been obtained for sharing of individual-level data; provide overall numbers in this Reporting Summary. Please state if this information has not been collected. Report sex- and gender-based analyses where performed, justify reasons for lack of sex- and gender-based analysis.*
Reporting on race, ethnicity, or other socially relevant groupings	*Please specify the socially constructed or socially relevant categorization variable(s) used in your manuscript and explain why they were used. Please note that such variables should not be used as proxies for other socially constructed/relevant variables (for example, race or ethnicity should not be used as a proxy for socioeconomic status). Provide clear definitions of the relevant terms used, how they were provided (by the participants/respondents, the researchers, or third parties), and the method(s) used to classify people into the different categories (e.g. self-report, census or administrative data, social media data, etc.) Please provide details about how you controlled for confounding variables in your analyses.*
Population characteristics	*Describe the covariate-relevant population characteristics of the human research participants (e.g. age, genotypic information, past and current diagnosis and treatment categories). If you filled out the behavioural & social sciences study design questions and have nothing to add here, write "See above."*
Recruitment	*Describe how participants were recruited. Outline any potential self-selection bias or other biases that may be present and how these are likely to impact results.*
Ethics oversight	*Identify the organization(s) that approved the study protocol.*

Note that full information on the approval of the study protocol must also be provided in the manuscript.

Field-specific reporting

Please select the one below that is the best fit for your research. If you are not sure, read the appropriate sections before making your selection.

☒ Life sciences ☐ Behavioural & social sciences ☐ Ecological, evolutionary & environmental sciences

For a reference copy of the document with all sections, see nature.com/documents/nr-reporting-summary-flat.pdf

Life sciences study design

All studies must disclose on these points even when the disclosure is negative.

Sample size	No statistical method was used to predetermine sample size. Sample sizes were chosen based on commonly used group sizes in the field of AAV delivery and RBC hitchhiking, as well as on our previous studies demonstrating reproducible outcomes with similar numbers of biological replicates and animals. These sizes were considered sufficient to detect consistent and biologically meaningful differences while balancing resource use and ethical considerations.
Data exclusions	No data was excluded.
Replication	Reproducibility was confirmed by performing all key experiments with multiple independent biological replicates, and all findings were consistently reproduced. Some Supplementary experiments (Supplementary Fig. 1A, B, and Supplementary Fig. 16B) were designed as trend analyses or large-scale screens rather than for precise quantification; therefore, fewer replicates were used to conserve resources, while reproducibility was confirmed through consistency across groups and subsequent validation of the selected formulation conditions with full replication.
Randomization	Randomization was applied when possible, for example in animal studies. Mice were randomized prior to treatments to control for potential confounding factors such as litter effects, baseline physiological differences, and environmental (cage) variables. For in vitro experiments, allocation followed the experimental design (e.g., treatment vs. control), where formal randomization was not applicable.
Blinding	The manuscript contains data from both blinded and unblinded experiments. Blinding was applied where feasible (e.g., during image quantification and flow cytometry analysis) but not used in certain in vivo experiments where treatment groups were apparent to the investigator during injections and sample processing. In those cases, blinding was not possible, but the data were collected using objective quantitative assays (e.g., PCR, ELISA, flow cytometry), minimizing the risk of bias.

Reporting for specific materials, systems and methods

We require information from authors about some types of materials, experimental systems and methods used in many studies. Here, indicate whether each material, system or method listed is relevant to your study. If you are not sure if a list item applies to your research, read the appropriate section before selecting a response.

Materials & experimental systems

n/a	Involved in the study
☐	☒ Antibodies
☐	☒ Eukaryotic cell lines
☒	☐ Palaeontology and archaeology
☐	☒ Animals and other organisms
☒	☐ Clinical data
☒	☐ Dual use research of concern
☒	☐ Plants

Methods

n/a	Involved in the study
☒	☐ ChIP-seq
☐	☒ Flow cytometry
☒	☐ MRI-based neuroimaging

Antibodies

Antibodies used	CD326 (EpCAM), AF594, Bioss, Cat. BS-4889R-A594, clone unspecified, dilution 1:80.<br>CD31 (PECAM-1), BV711, eBioscience, Cat. 407-0311-82, clone 390, dilution 1:80.<br>CD45, BUV395, BD Biosciences, Cat. 564616, clone 104, dilution 1:80.<br>GFP, AF488, BioLegend, Cat. 338007, clone FM264G, dilution 1:160.<br>CD16/32, unconjugated, eBioscience, Cat. 14-0161-85, clone 93, dilution 1:30.
Validation	Anti-CD326 (AF594, Bioss, Cat. BS-4889R-A594): Validated in this study as part of the flow cytometry strategy (see Supplementary Methods).<br>Anti-CD31 (BV711, eBioscience, Cat. 407-0311-82, clone 390): Validated for flow cytometry (Supplementary Methods); manufacturer validation available at: https://www.thermofisher.com/antibody/product/CD31-PECAM-1-Antibody-clone-390-Monoclonal/407-0311-82<br>Anti-CD45 (BUV395, BD, Cat. 564616, clone 104): Validated for flow cytometry (Supplementary Methods); manufacturer validation available at: https://www.bdbiosciences.com/en-us/products/reagents/flow-cytometry-reagents/research-reagents/single-color-antibodies-ruo/buv395-mouse-anti-mouse-cd45-2.564616<br>Anti-GFP (AF488, BioLegend, Cat. 338007, clone FM264G): Validated for flow cytometry (Supplementary Methods); manufacturer validation available at: https://www.biolegend.com/en-gb/search-results/alexa-fluor-488-anti-gfp-antibody-9584?GroupID=BLG6275<br>Anti-CD16/32 (unconjugated, eBioscience, Cat. 14-0161-85, clone 93): Fc receptor blocking antibody; manufacturer validation available at: https://www.thermofisher.com/antibody/product/CD16-CD32-Antibody-clone-93-Monoclonal/14-0161-82

Eukaryotic cell lines

Policy information about cell lines and Sex and Gender in Research

Cell line source(s)	EA.hy926 was purchased from ATCC (cat no. CRL-2922)<br>Primary human endothelial cells are from Cell Systems (cat no. ACBRI376; lot no. 376.03.05.01.2F; pediatric male doner)
Authentication	EA.hy926 was not authenticated.
Mycoplasma contamination	EA.hy926 was not tested for mycoplasma contamination.
Commonly misidentified lines<br>(See ICLAC register)	The study did not involve any misidentified cell lines.

Animals and other research organisms

Policy information about studies involving animals; ARRIVE guidelines recommended for reporting animal research, and Sex and Gender in Research

Laboratory animals	Female C57BL/6 mice (aged 5–6 weeks for lung delivery studies, and 10 weeks for brain delivery)
Wild animals	The study did not involve use of wild animals.
Reporting on sex	There is no known mechanistic influence of sex on the functioning of the current system. The study used female mice.
Field-collected samples	The study did not involve use of field-collected samples.
Ethics oversight	All experiments were performed according to the approved protocols by the Institutional Animal Care and Use Committee (IACUC) of the Faculty of Arts and Sciences (FAS), Harvard University (protocol number 18-02-320-1).

Note that full information on the approval of the study protocol must also be provided in the manuscript.

Plants

Seed stocks

Report on the source of all seed stocks or other plant material used. If applicable, state the seed stock centre and catalogue number. If plant specimens were collected from the field, describe the collection location, date and sampling procedures.

Novel plant genotypes

Describe the methods by which all novel plant genotypes were produced. This includes those generated by transgenic approaches, gene editing, chemical/radiation-based mutagenesis and hybridization. For transgenic lines, describe the transformation method, the number of independent lines analyzed and the generation upon which experiments were performed. For gene-edited lines, describe the editor used, the endogenous sequence targeted for editing, the targeting guide RNA sequence (if applicable) and how the editor was applied.

Authentication

Describe any authentication procedures for each seed stock used or novel genotype generated. Describe any experiments used to assess the effect of a mutation and, where applicable, how potential secondary effects (e.g. second site T-DNA insertions, mosiacism, off-target gene editing) were examined.

Flow Cytometry

Plots

Confirm that:

☒ The axis labels state the marker and fluorochrome used (e.g. CD4-FITC).

☒ The axis scales are clearly visible. Include numbers along axes only for bottom left plot of group (a 'group' is an analysis of identical markers).

☒ All plots are contour plots with outliers or pseudocolor plots.

☒ A numerical value for number of cells or percentage (with statistics) is provided.

Methodology

Sample preparation

The lungs were harvested after perfusion and placed in PBS containing 1% FBS. The lungs were cut into small pieces (<1 mm), then digested and processed using a lung dissociation kit (Miltenyi, 130-095-927) into single cells. Briefly, digestion enzymes were added to the samples and then incubated at 37 °C for 30 minutes. The samples were placed on top of pre-wet 70 µm strainers, mashed with plungers, and rinsed with 10 mL PBS. The cell suspensions were collected in 50 mL tubes and centrifuged at 500 g for 5 min. The supernatant was removed, and the samples were washed twice with a cell staining buffer (BioLegend, 420201). If necessary, red blood cells were lysed with ACK buffer (Gibco, A1049201) before the washing step. The resulting cells were stained with antibodies. Briefly, sample pellets were resuspended with 20 µL of 1/30 diluted anti-CD16/32 antibody (Invitrogen, 14-0161-85) for 10 min at 4 °C to block Fc receptors. Next, 20 µL of the antibody cocktail containing aCD31, aCD326, and aCD45 was added and incubated for 25 min at 4 °C. For the cocktail, each antibody was diluted by 1/40, which was then diluted to a final dilution of 1/80 once mixed with the sample solution containing CD16/32-coated cells. After the incubation, the samples were washed twice with PBS at 250 g for 5 min via centrifugation, followed by live/dead staining with LIVE/DEAD Fixable Blue (Invitrogen, L23105). Next, the samples were fixed and permeabilized using the BD perm/fix kit (BD, 554714) following the manufacturer's protocol for intracellular staining. Anti-GFP antibody was diluted by 1/160 for staining. After the staining, samples were washed twice with the perm/wash buffer, then finally resuspended into 200 µL of the staining buffer and stored at 4 °C until analysis.

Instrument

Cytek® Aurora

Software

FlowJo 10.10.0

Cell population abundance

The lungs were processed into single cells using Miltenyi Biotec's mouse lung dissociation kit, and the samples did not go through a sorting process for purification. A fraction of the sample was used to run through a flow cytometer, giving enough numbers (>50,000) of each cell type (endothelial & epithelial cells) for analysis.

Gating strategy

Briefly, 1. cells were broadly gated based on FSC-A vs. SSC-A, 2. gated on singlets based on SSC-H vs. SSC-A, 3. live non-leukocytes were gated as CD45-Live Dead Blue-, then 4. endothelial and epithelial cells were identified based on CD45-CD31+ and CD45-CD326+, respectively. 5. Each cell type was plotted as GFP vs. SSC-A to gate on GFP+ populations using non-AAV-treated samples as negative controls.

☒ Tick this box to confirm that a figure exemplifying the gating strategy is provided in the Supplementary Information.
